# Scrub Typhus in the Republic of Palau, Micronesia

**DOI:** 10.3201/eid1010.040288

**Published:** 2004-10

**Authors:** A. Mark Durand, Stevenson Kuartei, Ishmael Togamae, Maireng Sengebau, Linda Demma, William Nicholson, Michael O'Leary

**Affiliations:** * Department of Health Services, Yap State, Colonia, Yap, Federated States of Micronesia;; † Ministry of Health, Koror, Republic of Palau;; ‡ Centers for Disease Control and Prevention, Atlanta, Georgia, USA,; § Pacific Islands Health Officer Association, Agatna, Territory of Guam

**Keywords:** Pacific Islands, Micronesia, Palau, rickettsia, Orientia tsutsugamushi, Scrub typhus, dispatch

## Abstract

In October 2001, an outbreak of febrile illness began in the southwest islands group of the Republic of Palau. Through October 2003, a total of 15 southwest islanders experienced fever >39.5°C and abdominal distress, both lasting >7days. *Orientia tsutsugamushi*, the agent of scrub typhus, was subsequently identified as the cause.

Scrub typhus, a rickettsial disease caused by *Orientia tsutsugamushi*, is spread by biting larval trombiculid mites. Geographically specific foci of scrub typhus are determined by the distribution of vector mites (*1*). Rodents of the family *Muridae* are also commonly infected with *O. tsutsugamushi*, and detecting antibodies to the organism in rodents provides evidence for human risk of acquiring the infection (*1**–**4*).

After an incubation period of 6 to 21 days, the infection manifests as a lengthy (5–36 days if untreated), nonspecific febrile illness, which is sometimes accompanied by gastrointestinal, respiratory, or central nervous system symptoms. Illness can be inapparent or severe. Death is reported to occur in 1% to 30% of untreated cases (*5**,**6*). Scrub typhus is endemic in the tropical and subtropical regions of the Asian continent, as well as in Indonesia, the Philippines, parts of Australia, Japan, northern China, the Russian far east, and Korea (*3**,**5**,**7**–**12*). After several decades of inactivity, the disease has recently been reported in the Torres Strait Islands of northern Australia and in the Maldive Islands (*13**,**14*). The disease has not previously been reported in Micronesia.

The Republic of Palau is an island nation in western Micronesia, 7° north of the equator and 900 km east of the Philippines (Figure). The nation comprises a cluster of main islands within a single outer reef, plus a group of low limestone islands that lie 300 km to the southwest (the southwest islands). Four of these southwest islands are inhabited. Numerous bird species also inhabit the islands. Humans, fruit bats, rats, pigs, and cats are the only resident mammals. Drinking water is collected in rain collection tanks. Residents of the islands farm small plots of land that are cleared from the forest. Each island has a dispensary with basic medications. The islands receive periodic visits from small fishing vessels from Indonesia and the Philippines.

**Figure:**
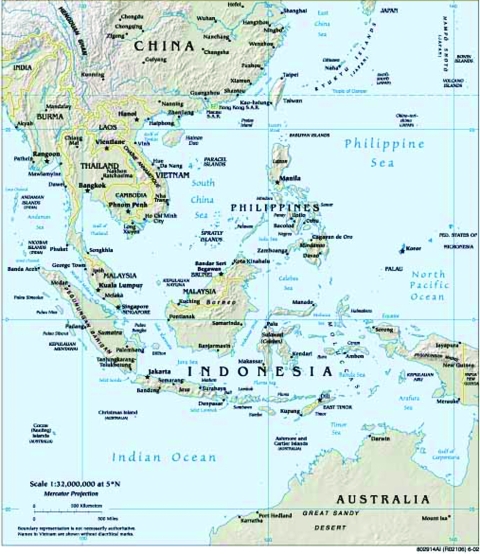
The Palau Islands. Map courtesy of The World Factbook, Central Intelligence Agency, 2004. http://www.cia.gov/cia/publications/factbook/geos/ps.html

From October 2001 to October 2003, 15 patients from the southwest islands required evacuation to the national hospital in the capital of Palau for treatment of a febrile illness, which was notable for temperature >39.5°C; duration >7 days (average, 12 days); and abdominal pain, vomiting, or both. Some patients had diarrhea, respiratory symptoms, headache, myalgia, and laboratory abnormalities as summarized in Table 1. Headache was described as severe, throbbing, and retroorbital. Sometimes headache was accompanied by photophobia. Rash was noted in 7 patients (47%). It typically appeared on day 4 or 5 of illness, first on the trunk, then spreading to the upper limbs, and lasted 1–3 days. Ten male and 5 female patients were affected. Their mean age was 15 years (range 3–58 years). The illness had no clear seasonal pattern. On two occasions, siblings residing together became ill at the same time. Patients did not appear to improve with empiric antimicrobial drug treatment administered in the field, which included ampicillin, cephalosporins, gentamicin, and metronidazole (none of which are known to be active against *O. tsutsugamushi*). Two patients had abdominal pain that prompted laparotomy; postsurgical diagnoses were mild appendicitis in one and ileitis in the other.

**Table 1:** Clinical findings among 15 scrub typhus patients in case series, Palau, 2001–2003

Finding	No. (%) of total patients, N = 15	No. (%) of sero-positive patients, N = 6
Fever	15 (100)	6 (100)
Abdominal pain	10 (67)	5 (84)
Vomiting	12 (80)	4 (67)
Headache	10 (67)	4 (67)
Rash	7 (47)	1 (16)
Cough/rhinorrhea	6 (40)	2 (33)
Elevated alanine aminotransferase	6 (40)	2 (33)
Proteinuria	4 (27)	1 (16)
Myalgia	3 (20)	2 (33)
Conjunctival suffusion	3 (20)	2 (33)
Leukocytosis	3 (20)	2 (33)
Diarrhea	3 (20)	1 (16)
CSF pleocytosis	2 (13)	0
Visible blood in stool	1 (7)	0

^a^CSF, cerebrospinal fluid.

All but 1 of the 15 patients came from a single island, Sonsoral, which has only 40 inhabitants (Figure). The other lived on the island of Tobi (with 35 inhabitants), approximately 100 miles from Sonsoral, and had not left that island for several years before the onset of illness.

Standard cultures and serologic tests (including those for dengue virus, *Leptospira*, and hepatitis virus), available at the national hospital did not indicate the cause of the illness; however, all patients had received antimicrobial drugs before cultures could be taken. In April 2003, paired serum specimens from one patient that were sent to a commercial reference laboratory in Hawaii had negative results for *Salmonella typhi* and *Rickettsia prowazekii*. At the request of the Ministry of Health, the Centers for Disease Control and Prevention (Atlanta, GA) performed serologic testing of specimens collected from six patients. Results were negative or indeterminate for typhoid fever (by Typhidot IgG and IgM and Tubex (Typhidot, Malaysian Biodiagnostic Research SDN BHD, Kuala Lumpur, Malaysia). Each serum sample was tested for antibodies to *O. tsutsugamushi* by indirect immunofluorescence assay (IFA) after the method of Elisberg and Bozeman (*15*). Antigen suspensions from the Karp strain of *O. tsutsugamushi* were prepared in chicken yolk sac, and vials of antigen suspension at optimal dilution were frozen at –75°C. The antigen suspension was pipetted onto slides coated with bovine serum albumin (BSA, 1%), air dried, fixed with acetone, and stored at –75°C until use. Slides were warmed to room temperature in desiccated conditions. Serial twofold dilutions, beginning at 1/16, were made in sample diluent (phosphate-buffered saline [PBS], pH 7.38 with 1% BSA and 1% normal goat serum). For the initial screening, two dilutions (1/16 and 1/256) were added to slides and incubated for 30 min at 37°C, followed by washing in PBS, pH 7.38, for 15 min (3 washes x 5 min). An optimized dilution (1/150) of fluorescein isothiocyanate (FITC)-labeled goat antihuman conjugate immunoglobulin (Ig) G (g-chain-specific) (Kirkegaard and Perry Laboratories, Inc., Gaithersburg, MD) was then applied to the slides, which were incubated and washed as before, except that eriochrome black T counterstain was added to the middle wash. Glycerol-PBS mounting medium was added to each well, a coverslip was applied, and the slides were read at a magnification of 400x with an epifluorescence UV microscope. Any reactive samples were then titrated to endpoint by using IgG-specific (g) conjugate. Titers were recorded as the reciprocal of the highest dilution displaying specific fluorescence.

For IgM testing, the samples were first depleted of IgG by using a recombinant Protein G device (Rapi-Sep-M kit, Pan Bio, Columbia, MD). This procedure resulted in a final 1/8 dilution of the serum sample. This solution was then diluted further in sample diluent and placed onto slides. The protocol is similar to that detailed above, but it used FITC-labeled, goat antihuman IgM (m-chain specific) conjugate at a working dilution of 1/100. Only one serum specimen was available for two of the patients at day 10 and day 36 of their illness; paired serum specimens were available for the other four. The serum specimens of all six patients had high titers of antibodies to *O. tsutsugamushi*. (Table 2).

**Table 2:** *Orientia tsutsugamushi* IgG and IgM antibody titers for six southwest islanders with prolonged fever and abdominal distress^a^

Patient no.	Antibody type	Acute-phase titer	Convalescent-phase titer
1	IgG	1:2,048	NA
	IgM	1:16,384	NA
2	IgG	NA	1:32,768
	IgM	NA	1:2,048
3	IgG	1:262,144	1:262,144
	IgM	1:4,096	1:1,024
4	IgG	1:65,536	1:65,536
	IgM	1:1,024	1:2,048
5	IgG	1:8,000	1:64,000
	IgM	NA	NA
6	IgG	1:4,000	1:64,000
	IgM	NA	NA

^a^Ig, immunoglobulin; NA, result not available.

In this outbreak of scrub typhus in the southwest islands of the Republic of Palau, abdominal distress was a prominent feature. However, none of our patients had an inoculation-site eschar, including the two patients who were examined after we became aware of the disease in the southwest islands. The eschar associated with scrub typhus can have minimal symptoms and be hidden within skin folds or hairy body areas. The absence of eschar has been noted previously in Southeast Asian patients (*11*). Although no deaths occurred in this outbreak, the cases were sufficiently severe to require evacuation by boat, a difficult and expensive measure that is taken only in cases of life-threatening illness in Palau. Sonsoral, the island with 14 of the cases, has a population of 40 residents; thus, the attack rate for symptomatic disease on Sonsoral was 35%, higher than has previously been reported for this disease.

After this cluster of scrub typhus cases was recognized, a campaign to educate the local community about the disease was launched in the southwest islands and in the capitol. Controlling the rat population, wearing clothing and using repellants when in contact with grass and brush, and eliminating brush near households were emphasized. The public and healthcare workers are also taught the importance of early recognition and antimicrobial drug treatment of possible patients.

Important questions remain regarding the reasons for the high attack rate on Sonsoral, whether scrub typhus is newly introduced in the region or only newly recognized, and the distribution and dynamics of *O. tsutsugamushi*, its vector, reservoir(s), and human hosts in Palau and elsewhere in Micronesia. Serologic assessments and studies of the local environment are needed to clarify these issues.
