# Correction, vol. 10, no. 10

**DOI:** 10.3201/eid1011.C11011

**Published:** 2004-11

**Keywords:** errata, erratum, correction

In "Scrub Typhus in the Republic of Palau, Micronesia," by A. Mark Durand et al. (p. 1840), Table 2 was incorrect. The correct version appears below and online at http://wwwnc.cdc.gov/eid/article/10/10/04-0288_article.htm

**Table 2:** *Orientia tsutsugamushi* IgG and IgM antibody titers for six southwest islanders with prolonged fever and abdominal distress^a^

Patient no.	Antibody type	Acute-phase titer	Convalescent-phase titer
1	IgG	1:2,048	NA
	IgM	1:16,384	NA
2	IgG	NA	1:32,768
	IgM	NA	1:2,048
3	IgG	1:262,144	1:262,144
	IgM	1:4,096	1:1,024
4	IgG	1:65,536	1:65,536
	IgM	1:1,024	1:2,048
5	IgG	1:8,000	1:64,000
	IgM	NA	NA
6	IgG	1:4,000	1:64,000
	IgM	NA	NA

^a^Ig, immunoglobulin; NA, result not available.

We regret any confusion this error may have caused.

